# Effectiveness of a group nursing intervention in reducing anxiety and depression for smoking cessation: a quasi-experimental study in light of Afaf Meleis’ Transition Theory

**DOI:** 10.17533/udea.iee.v44n1e10

**Published:** 2026-03-30

**Authors:** Isla Daniela Da Silva Pinto, Raphael Alves da Silva, Yure Rodrigues Silva, Emanuela Batista Ferreira e Pereira, Felipe Almeida Sales, Carla Aparecida Arena Ventura, Fagner Alfredo Ardisson Cirino Campos, Felicialle Pereira da Silva

**Affiliations:** 1 Nurse, Master’s. Email: isladaniela.pinto@upe.br https://orcid.org/0000-0003-4703-979X Universidade de Pernambuco Brazil isladaniela.pinto@upe.br; 2 Nurse, Resident. Email: raphael.alvess@upe.br https://orcid.org/0000-0003-2555-909X Instituto de Medicina Integral Professor Fernando Figueira Brazil raphael.alvess@upe.br; 3 Nurse, Master’s. Email: yurerodrigues.enf@gmail.com https://orcid.org/0009-0003-0299-394X Universidade de Pernambuco Brazil yurerodrigues.enf@gmail.com; 4 Nurse, PhD. Professor. Email: emanuela.pereira@upe.br https://orcid.org/0000-0003-4665-4379 Universidade de Pernambuco Brazil emanuela.pereira@upe.br; 5 Nurse, Master’s. Email: felipealmeida.sales@upe.br https://orcid.org/0009-0008-5229-8087 Universidade de Pernambuco Brazil felipealmeida.sales@upe.br; 6 Bachelor of Laws. Ph.D. Full Professor. Email: caaventu@eerp.usp.br https://orcid.org/0000-0003-0379-913X Universidade de São Paulo Brazil caaventu@eerp.usp.br; 7 Nurse, PhD. Candidate. Email: fagnerardisson@usp.br https://orcid.org/0000-0001-6563-6155 Universidade de São Paulo Brazil fagnerardisson@usp.br; 8 Nurse, PhD. Professor. Email: felicialle.pereira@upe.br https://orcid.org/0000-0002-2805-7506 Universidade de Pernambuco Brazil felicialle.pereira@upe.br; 9 Nossa Senhora das Graças School of Nursing, University of Pernambuco (UPE), Recife, Brazil. Universidade de Pernambuco Nossa Senhora das Graças School of Nursing University of Pernambuco (UPE) Recife Brazil; 10 Instituto de Medicina Integral Professor Fernando Figueira (IMIP), Hospital Otávio de Freitas, Recife, Pernambuco, Brazil. Instituto de Medicina Integral Professor Fernando Figueira Instituto de Medicina Integral Professor Fernando Figueira (IMIP) Hospital Otávio de Freitas Recife Pernambuco Brazil; 11 Department of Psychiatric Nursing and Human Sciences, University of São Paulo (USP), São Paulo, Brazil Universidade de São Paulo Department of Psychiatric Nursing and Human Sciences University of São Paulo (USP) São Paulo Brazil

**Keywords:** smoking cessation, anxiety, depression, support group, nursing theory., cese del hábito de fumar, ansiedad, depresión, grupos de autoayuda, teoría de enfermería., abandono do hábito de fumar, ansiedade, depressão, grupo de apoio, teoria de enfermagem.

## Abstract

**Objective.:**

To evaluate the effectiveness of a group intervention for smoking cessation in reducing anxiety and depression symptoms in individuals who stop using nicotine, based on Afaf Meleis’ Transition Theory.

**Methods.:**

A longitudinal, quasi-experimental study with a convenience sample of 15 volunteers. The intervention was led by a qualified nurse and structured in two stages: an intensive phase with weekly sessions during the first three months (total of 12 sessions) and a maintenance phase with biweekly sessions from the fourth to the sixth month (total of 6 sessions). Data was collected at three time points: baseline (before the intervention), three months later, and at the end of six months from the start of the intervention. The following instruments were used: the Patient Health Questionnaire-9, the State-Trait Anxiety Inventory, and the Fagerström Test for Nicotine Dependence.

**Results.:**

A statistically significant reduction in nicotine dependence was observed throughout the study. The percentage of “very high” dependence decreased from 46.7% to 6.7% between the first and third assessment points. A significant decrease in trait anxiety was observed (p = 0.028), while no significant differences were found in depressive symptoms across the three assessment times. At the third assessment, a positive correlation was identified between the level of dependence and situational anxiety levels.

**Conclusion.:**

The group intervention studied had a positive impact on the smoking cessation process, reflected in reduced nicotine dependence and lower anxiety levels, highlighting the importance of collective support in coping with dependence.

## Resumen

**Objetivo.:**

Evaluar la eficacia de una intervención grupal para la cesación tabáquica en la reducción de los síntomas de ansiedad y depresión, fundamentada en la Teoría de las Transiciones de Afaf Meleis.

**Métodos.:**

Estudio longitudinal, cuasi-experimental, con una muestra por conveniencia de 15 voluntarios. La intervención fue liderada por una enfermera calificada y estuvo estructurada en dos etapas: una fase intensiva con sesiones semanales durante los primeros tres meses (total de 12 sesiones) y una fase de mantenimiento con sesiones quincenales del cuarto al sexto mes (total de 6 sesiones). La recolección de datos ocurrió en tres momentos: línea de base (antes de la intervención), a los tres meses y al final de los seis meses desde el inicio de la intervención. Se utilizaron el Cuestionario de Salud del Paciente-9, el Inventario de Ansiedad Rasgo-Estado e el Test de Fagerström para la Dependencia de la Nicotina.

**Resultados.:**

Se observó una reducción estadísticamente significativa en la dependencia a la nicotina; el nivel "muy elevado" disminuyó del 46.7% al 6.7% entre el primer y tercer momento. Hubo una disminución significativa en la ansiedad-rasgo (p=0.028), sin diferencias significativas en los síntomas depresivos. En la tercera evaluación, se identificó una correlación positiva entre el grado de dependencia y la ansiedad-estado.

**Conclusión.:**

La intervención coordinada por enfermería, como agente facilitador de la transición, tuvo un impacto positivo en la reducción de la dependencia y la ansiedad, reforzando la importancia del apoyo colectivo estructurado para la sostenibilidad de la cesación.

## Resumo

**Objetivo.:**

Avaliar a eficácia de uma intervenção em grupo para cessação tabágica na redução dos sintomas de ansiedade e depressão em indivíduos que param de usar nicotina, com base na teoria da transição de Afaf Meleis.

**Métodos.:**

Estudo longitudinal, quase-experimental, com amostra por conveniência de 15 voluntários. A intervenção foi liderada por enfermeira qualificada e estruturada em duas etapas: fase intensiva com sessões semanais durante os primeiros três meses (total de 12 sessões) e fase de manutenção com sessões quinzenais do quarto ao sexto mês (total de 6 sessões). A coleta de dados ocorreu em três momentos: linha de base (antes da intervenção), aos três meses seguintes e ao final dos seis meses do início da intervenção. Foram utilizados o Questionário de Saúde do Paciente-9, o Inventário de Ansiedade Traço-Estado e o Teste de Fagerström para Dependência de Nicotina.

**Resultados.:**

Observou-se uma redução estatisticamente significativa na dependência de nicotina ao longo do estudo. A porcentagem de dependência "muito alta" diminuiu de 46.7% para 6.7% entre o primeiro e o terceiro momento de avaliação. Observou-se uma diminuição significativa na ansiedade traço (p=0.028), enquanto não foram observadas diferenças significativas nos sintomas depressivos entre os três momentos de avaliação. Na terceira avaliação, foi identificada uma correlação positiva entre o grau de dependência e os níveis de ansiedade situacional.

**Conclusão.:**

A intervenção estudada no grupo obteve impacto positivo no processo de cessação tabágica, o que se refletiu na redução da dependência de nicotina e dos níveis de ansiedade, destacando a importância do apoio coletivo no enfrentamento da dependência.

## Introduction

Smoking consists of the habitual use of tobacco-derived products, such as nicotine, a psychoactive substance with a high capacity to generate physical and psychological dependence by acting on brain reward circuits.^1^ According to the World Health Organization (WHO), the global prevalence of smokers in 2023 was 16.4%, corresponding to approximately 1.3 billion people.^2^ In Brazil, recent data indicate a reversal in the previous downward trend, with the smoking rate increasing from 9.3% in 2023 to 11.6% in 2024.^3^ It is estimated that by 2030, eight million deaths per year will be attributable to tobacco, with more than 80% of these occurring in low- and middle-income countries.^2^^,^^4^ Nationally, smoking is associated with 12.6% of deaths among individuals over 35 years old, totaling more than 156,000 potentially preventable deaths annually.^5^

In addition to physical risks, smoking has a significant impact on mental health. Individuals with nicotine dependence are two to eight times more likely to develop anxiety and depressive disorders compared to non-smokers or occasional smokers. A meta-analysis involving more than 500,000 participants confirmed that smokers have a 24% higher risk of developing depressive symptoms.^5^ In Brazil, observational studies support this association, indicating that smokers have an odds ratio 2.01 times higher for anxiety symptoms and 4.80 for depressive symptoms.^6^ Despite this complex burden of morbidity, smoking is a preventable condition and remains a priority target for public health interventions.^7^^,^^8^ In this context, smoking cessation is understood as a health transition process which, according to Afaf Meleis, involves significant changes in life conditions and requires personal, emotional, and social adaptation. Quitting smoking is specifically characterized as a complex situational transition: individuals are challenged to break deeply rooted habitual behaviors while developing new self-care patterns.^8^ Given this complexity, public policies emerge as essential facilitating agents. In Brazil, the Ministry of Health and the National Cancer Institute (INCA) have consolidated, since the 1980s, the National Tobacco Control Program (NTCP). 

This program adopts a logical model integrating educational, legislative, and healthcare actions aimed at supporting cessation and reducing tobacco-related morbidity and mortality.^9^

Evidence shows that group interventions are more effective than brief individual counseling because they foster social support, mutual identification, and coping strategies-key pillars for successful situational transitions. However, in Latin America, the effectiveness of this process faces challenges such as the high prevalence of psychiatric comorbidities and disparities in access to comprehensive primary care support. Overcoming these gaps therefore requires models that integrate behavioral change with the management of emotional symptoms in order to reduce the regional burden of morbidity and mortality. Although the NTCP provides a robust care framework, uncertainties remain regarding how interventions grounded in nursing theories can optimize outcomes. In this context, the present study is guided by the following research question: what is the effect of a group intervention, based on Transition Theory, on reducing nicotine dependence and symptoms of anxiety and depression among smokers?

## Methods

### Type of study

This study has a quasi-experimental, longitudinal design, following the recommendations of the Transparent Reporting of Evaluations with Nonrandomized Designs (TREND) to promote transparent and standardized reporting of results.^10^

### Setting and period

The research took place in a referral polyclinic linked to the municipal health network of Recife, Pernambuco, recognized for its specialized support for smoking cessation. Activities occurred between January and August 2024, encompassing recruitment and longitudinal monitoring phases.

### Population and sample

The sample was selected through non-probabilistic convenience sampling, including individuals over 18 years old who attended at least 75% of the intervention sessions. Previously, a pilot study was conducted with the first five participants to test instrument applicability and intervention logistics. Since no adjustments to the methodological protocol were required, these participants were included in the final sample analysis.

### Data collection

*1) Participant selection* **:** Recruitment occurred sequentially from the unit’s waiting list until reaching a total of 15 participants, according to Brazilian Ministry of Health parameters for group interventions.^11^ Individuals with cognitive limitations that could impair understanding of the scales were excluded. The intervention was conducted by a nurse properly trained by INCA, who, prior to group activities, carried out an individual anamnesis in a private setting, lasting a mean of 25 minutes, to collect participants’ sociodemographic and clinical profiles. At this stage, smoking history, comorbidities, and motivational factors for smoking and cessation were investigated. *2) Evaluation protocol (longitudinal):* The intervention followed the NTCP/INCA protocol, with 90-minute sessions focused on a cognitive-behavioral approach, structured into two phases: an intensive phase with 12 weekly sessions during the first quarter, and a maintenance phase with six biweekly sessions from the fourth to the sixth month. Longitudinal monitoring included data collection at three time points: baseline (pre-intervention), at the end of the third month, and at the sixth month after protocol initiation. To ensure internal validity, each phase’s assessments were completed within a maximum interval of one week.

### Theoretical framework

In light of Afaf Meleis’ Transitions Theory,^8^ the intervention was designed to act as a transition-facilitating agent. The nurse, as group leader, mediated transition properties (engagement and search for new meanings), providing targeted support for managing dependence and emotional symptoms, aiming at healthy adaptation to a tobacco-free lifestyle.

### Data collection instruments

1)To characterize participants’ profiles, a structured sociodemographic questionnaire was used, including variables such as age, sex, marital status, education, employment status, and income. These data enabled contextualization of factors influencing smoking cessation and analysis of patterns associated with mental health outcomes; 2) Depressive symptoms were assessed using the Patient Health Questionnaire-9 (PHQ-9), validated in Brazil for major depression screening. The PHQ-9 consists of nine items based on the diagnostic criteria of the Diagnostic and Statistical Manual of Mental Disorders (DSM-V).^12^ Each item evaluates symptom frequency over the previous two weeks, with total scores ranging from 0 to 27. The Brazilian version has robust psychometric properties, with a Cronbach’s alpha of 0.85 in national validation;^13^ 3) Anxiety symptoms were measured using the State-Trait Anxiety Inventory (STAI), translated and adapted into Portuguese by Biaggio. The instrument evaluates anxiety in two dimensions: trait anxiety (stable predisposition/personality trait) and state anxiety (transient emotional reaction). It contains 40 items equally divided between the two subscales and is recognized for clinical validity and reliability in the Brazilian context;^14^ and, 4) Physical nicotine dependence was assessed using the Fagerström Test for Nicotine Dependence (FTND). Composed of six items with scores ranging from 0 to 10, it is the gold standard recommended by (NTCP)/INCA. In the literature, the FTND shows internal consistency coefficients between 0.55 and 0.65 and is effective for stratifying dependence levels to guide therapeutic management.^15^

### Data analysis

Initially, data were entered into Microsoft Excel® spreadsheets and later analyzed using IBM SPSS® software, version 27, adopting a 5% significance level (*p* < 0.05). Data were analyzed descriptively using absolute and percentage frequencies for categorical and ordinal variables, while numerical variables were summarized using measures of central tendency and dispersion (mean, median, and standard deviation). Comparisons among the three evaluation time points were performed using the Friedman test for ordinal-scale variables and Cochran’s Q test for dichotomous categorical variables. When statistically significant differences were identified, post-hoc tests (multiple comparisons) were conducted to determine which phase pairs differed.

### Group operationalization

The group was conducted through structured sessions with a defined program. The methodology used multimodal resources (support texts, videos, music, and participation of invited professionals) to diversify stimuli and promote engagement. Topics were addressed sequentially and progressively, including: impacts on physical and mental health; identification of triggers; cognitive-behavioral strategies for craving management; strengthening emotional support; and relapse prevention. Each meeting followed a standardized four-stage script: Welcoming (individualized attention to participants); Educational Action (presentation of theoretical-practical information); Group Interaction (collective review and discussion, where sharing experiences and achievements fostered new meanings about the transition process); and Home Assignments (focused on practical application of content). Sessions were guided by the manual *“Quitting Smoking without Mysteries”*, ensuring standardization of the group intervention.^9^

### Ethical aspects

 The study was approved by the Research Ethics Committee of the University of Pernambuco, under approval opinion number 6,587,065 and CAAE 76437323.7.0000.5192, in compliance with all ethical principles established by National Health Council Resolution 466/2012. All participants were duly informed about the study’s objectives, risks, and benefits and signed an informed consent form prior to data collection.

## Results

The study sample consisted of 15 individuals aged between 33 and 66 years, with a mean age of 49.87 years (SD = 10.03) and a median of 48 years. Regarding sociodemographic characteristics, Table 1 shows a predominance of females (66.7%) and participants who were born in or came from Recife (80%). Most identified as heterosexual (86.7%), of mixed race (66.7%), and Catholic (60%). Among the participants, 53% earned between 1 and 3 minimum wages per month, corresponding to a range between US$285 and US$855 in 2024. The most frequent education level was completion of high school (40%), followed by completion of university education (20%).

In the assessment of participants’ health profiles, 40% were found to have a previously established clinical diagnosis. Among the most frequently reported conditions, systemic arterial hypertension stood out, mentioned by 13.3% of individuals. Other comorbidities were identified, including diabetes mellitus, gastritis, attention deficit hyperactivity disorder, and heart diseases, each with a frequency of 6.7%. Among the study participants, only one began using tobacco in adulthood; the others started smoking during adolescence, at ages ranging from 11 to 20 years.

Regarding therapeutic support for cessation, it was observed that the majority of participants, n=12 (80.0%), used adjuvant pharmacological intervention alongside group therapy. The most prevalent pharmacological combination was the use of Bupropion Hydrochloride associated with a nicotine patch, observed in n=8 (53.3%) of cases. Other modalities included the isolated use of a nicotine patch, n=2 (13.3%), and the triple combination of Bupropion Hydrochloride, patch, and nicotine gum, n=2 (13.3%). Only n=3 (20%) of participants completed the cessation process exclusively through group behavioral intervention, without pharmacological support.

Table 2 presents the evolution of the degree of nicotine dependence among participants in the three phases of data collection, as assessed by the Fagerström Test. A significant reduction in dependence levels was observed over time. In the first assessment, 46.7% of participants presented very high dependence and 26.7% high dependence. In the third phase, these percentages decreased to 6.7% in both categories. Statistical analysis revealed a significant difference between the evaluated time points (p<0.001), indicating a statistically relevant reduction in nicotine dependence associated with the intervention performed during the group.

**Table 1:** Sociodemographic characteristics of the 15 participants in the smoking cessation group

Variables	***n* (%)**
Sex	
Male	5 (33.3)
Female	10 (66.7)
Origin	
Recife	12 (80.0)
Olinda	1 (6.7)
Timbaúba	1 (6.7)
Rio de Janeiro	1 (6.7)
Sexual orientation	
Heterosexual	13 (86.7)
Homosexual	2 (13.3)
Race/color	
White	3 (20.0)
Brown	10 (66.7)
Black	2 (13.4)
Marital status	
Married	8 (53.3)
Divorced	5 (33.3)
Single	2 (13.3)
Education	
Incomplete elementary education	4 (26.7)
Elementary education	2 (13.3)
High school	6 (40.0)
University education	3 (20.0)
Religion	
Catholic	9 (60.0)
No religion	6 (40.0)
Income (minimum wage)*	
Less than 1	3 (20.0)
One to three	8 (53.3)
Greater than 3	4 (26.7)

* One minimum wage is equivalent to US$288.00

**Table 2:** Assessment of the degree of nicotine dependence of the 15 participants in the smoking cessation group

Fagerström	Time lapse of data collection		
	**First *n* (%)**	**Second *n* (%)**	**Third *n* (%)**
Very low dependence	-	7 (46.7)	9 (60.0)
Low dependence	3 (20.0)	4 (26.7)	2 (13.3)
Medium dependence	1 (6.7)	2 (13.3)	2 (13.3)
High dependence	4 (26.7)	1 (6.7)	1 (6.7)
Very high dependence	7 (46.7)	1 (6.7)	1 (6.7)
Total	15 (100.0)	15 (100.0)	15 (100.0)

Table 3 presents the results regarding trait and state anxiety levels, as assessed by the State-Trait Anxiety Inventory (STAI), in the three phases of data collection. A reduction in scores was observed in both dimensions of anxiety throughout the monitoring, with the decrease in trait anxiety being statistically significant (p = 0.028). The third phase stands out, in which 71.4% of participants presented low levels of state anxiety, demonstrating a significant improvement in the control of emotional responses to everyday situations.

**Table 3:** Assessment of trait anxiety and state anxiety among participants in the smoking cessation group

Level of anxiety	Time lapse of data collection			***p-value***
	**First *n* (%)**	**Second *n* (%)**	**Third *n* (%)**	
State				0.166
Low	7 (46.7)	6 (40.0)	11 (73.3)	-
Medium	7 (46.7)	9 (60.0)	4 (26.7)	-
High	1 (6.7)	-	-	-
Trace	-	-	-	0.028
Low	2 (13.3)	9 (60.0)	7 (46.7)	-
Medium	11 (73.3)	5 (33.3)	8 (53.3)	-
High	2 (13.3)	1 (6.7)	-	-
Total	15 (100.0)	15 (100.0)	15 (100.0)	-

Table 4 presents the results of the assessment of depressive symptoms using the PHQ-9 throughout the three phases of data collection. It was observed that the proportion of participants without depressive symptoms remained constant (53.3%) throughout the study period, indicating stability in this subgroup. 

On the other hand, 46.7% of participants presented some level of depression. Statistical analysis did not identify significant differences between the three assessment points (p=0.67), suggesting that the variations in depression scores were not sufficiently consistent to indicate relevant changes over time.

**Table 4:** Assessment of depression levels according to PHQ-9 of participants in the smoking cessation group

PHQ-9	Time lapse of data collection		
	**First *n* (%)**	**Second *n* (%)**	**Third *n* (%)**
No depression	8 (53.3)	8 (53.3)	8 (53.3)
Mild depression	1 (6.7)	1 (6.7)	1 (6.7)
Moderate depression	1 (6.7)	1 (6.7)	1 (6.7)
Moderately severe depression	4 (26.7)	3 (20.0)	5 (33.3)
Severe depression	1 (6.7)	2 (13.3)	0 (0.0)
Total	15 (100.0)	15 (100.0)	15 (100.0)

Table 5 presents the correlations between Fagerström Test scores and state anxiety levels assessed in the three phases of data collection. In the third phase, a statistically significant correlation was identified between the degree of nicotine dependence and state anxiety levels, with a positive association. This indicates that, even after the group intervention, participants with higher levels of state anxiety tended to maintain higher scores of nicotine dependence, suggesting a relationship between momentary emotional state and difficulty in smoking cessation.

**Table 5:** Spearman correlations of the Fagerström scale with state anxiety, trait anxiety, and depression, according to the data collection phase

Variables	Time lapse of data collection		
	First *r* (*p*-value)	Second *r* (*p*-value)	Third *r* (*p*-value)
Anxiety state	0.29 (0.287)	0.50 (0.071)	0.63 (0.015)
Anxiety trace	0.00 (1.000)	0.31 (0.280)	0.44 (0.118)
Depression	0.15 (0.589)	0.22 (0.442)	0.11 (0.691)

## Discussion

This study revealed a predominance of females among participants, a finding that corroborates previous investigations, which also identified greater adherence of women to smoking cessation groups.^16^^,^^17^ This trend reflects the more proactive behavior of women in seeking assistance, accessing health services more regularly. However, although women show greater engagement in programs, the global prevalence of smoking remains higher among men.^18^ From Meleis’ perspective, social and cultural factors shape the properties of transition. Greater female adherence suggests a higher level of preparedness for quitting smoking, whereas men may face gender-related barriers that hinder mobilization and entry into the transition process.^8^

The mean age of smoking initiation among participants occurred during adolescence, a critical period for tobacco experimentation mediated by curiosity and peer influence. From Meleis’ theoretical framework, the onset of smoking can be interpreted as a transition in which environmental and community factors strongly influence the individual’s experience. Meleis argues that such structural and social conditions impact both the risk and the response to transition. Therefore, although the context facilitated the beginning of the habit, structured professional support acts as a facilitating agent that allows individuals to reframe this trajectory and pursue smoking cessation later in life.

Additionally, the fact that tobacco is a legal drug contributes to greater access and social acceptance, especially among peers, which may facilitate the onset of use during adolescence. These data are consistent with the literature, which points to social influences as one of the main motivating factors for smoking initiation.^21^ It was found that nearly half of the participants had some comorbidity, including heart disease, diabetes, and gastrointestinal disorders. These conditions are recognized in the literature for their association with smoking, which acts not only as a risk factor for their development but also as an aggravating element, contributing to worse clinical outcomes. The consumption of tobacco-derived products is directly related to increased morbidity rates and higher premature mortality in the affected population.^22^

The literature shows that smoking worsens the prognosis of chronic diseases such as *diabetes mellitus* and hypertension by enhancing systemic inflammation and oxidative stress, thereby increasing the risk of cardiovascular and renal events.^23^^,^^24^ In this context of clinical vulnerability, the progressive reduction in nicotine dependence levels observed throughout the group sessions reinforces the effectiveness of the collective approach. This decline in physical dependence corroborates recent findings demonstrating how continuous participation in group activities can reverse initially high-dependence profiles.^24^

Due to the severity of baseline dependence, most participants used pharmacological support (Bupropion and nicotine replacement), a practice aligned with the literature recommending combined therapy to optimize outcomes in complex cases.^25^ From Meleis’ perspective, this multidimensional approach is fundamental, since an effective transition requires interventions that integrate physiological, emotional, and social aspects.^8^ By recognizing the complexity of this process, the group intervention promoted preparation and engagement, facilitating the strengthening of a new tobacco-free identity and mitigating the psychological distress inherent to transition.

The smoking cessation rate observed in this study was 53.3%, a result demonstrating the effectiveness of the group intervention. Other studies have evaluated the outcomes of these protocols adopted in public health systems across different regions of Brazil and worldwide, considering success rates immediately after completion of the four recommended sessions. Consistent with the present findings, another investigation also reported cessation rates above 50%. All participants showed some level of anxiety, reinforcing the association between smoking and anxious symptoms. Data analysis revealed distinct patterns among the evaluated dimensions: while state-anxiety levels tended to decrease over time, trace-anxiety levels showed an even more pronounced reduction. This difference may indicate that although the group provided momentary relief from emotional responses associated with specific situations (state-anxiety), it also contributed to more lasting changes in the predisposition pattern to anxiety (trace-anxiety). Difficulty controlling anxiety represents one of the main barriers to smoking cessation, since psychoactive compounds present in tobacco, such as nicotine, are related to the induction of pleasure and relaxation sensations, reinforcing use behavior and maintaining dependence.^21^^,^^22^ Stressful situations that trigger anxiety may act as relapse triggers by reinforcing maladaptive coping mechanisms in response to tension. Negative reinforcement contributes to the belief that “smoking is relaxing,” favoring the maintenance or resumption of smoking behavior.^23^

Researchers report that the maladaptive behavior of smoking, although harmful to health, can temporarily reduce stress and relieve anxiety, making cessation potentially challenging because it reinforces core beliefs that smoking is relaxing.^24^ In this context, the group acts as a facilitating intermediary, helping participants replace dysfunctional strategies. According to Meleis, transition occurs with internal demedicalization, openness to change, social support, and acquisition of new emotional and behavioral resources that promote physical and emotional relaxation.^8^

A positive correlation was identified between anxiety levels and the degree of nicotine dependence, showing that as participants progressed through treatment phases and gradually reduced cigarette consumption, there was also a significant reduction in anxious symptoms. From the perspective of Afaf Meleis’ Transitions Theory, smoking cessation is classified as a complex situational transition requiring individuals to redefine deeply rooted roles and behaviors. In this study, the statistically significant reduction in nicotine dependence and trace-anxiety functions as a positive process indicator, signaling that participants are developing effective coping strategies to manage change.

Contemporary nursing studies have used Transitions Theory to support interventions in mental health and substance dependence, demonstrating that group support acts as a fundamental nursing therapeutic strategy. Meleis considers it crucial that transition results in well-being; therefore, by sharing experiences in the group, smokers find what the theory calls support resources, essential to mitigate insecurity and anxiety, which are common during the transition from illness to self-care.^8^^,^^19^^,^^25^

This finding suggests that reducing dependence may contribute to anxiety relief, possibly due to the attenuation of withdrawal symptoms and the strengthening of emotional self-regulation throughout the cessation process. The literature reinforces this relationship: smokers are approximately twice as likely to present anxious symptoms compared to non-smokers, and reducing tobacco consumption is associated with decreased anxiety levels.^19^ Meleis highlights strengthened self-regulation as one of the criteria for successful transitions. The simultaneous reduction in nicotine dependence and anxiety levels indicates a healthy transition, as participants stop using tobacco as an emotional regulation strategy and adopt new coping methods.^8^

Based on PHQ-9 results, nearly half of the participants showed some degree of depression, ranging from mild to severe. Moderately severe depression scores fluctuated across research phases, decreasing in the second phase and increasing in the third, which may reflect individual variations in response to interventions as well as the influence of uncontrolled external factors. However, cases classified as severe depression were eliminated in the final phase, suggesting a possible positive response to the interventions conducted in the cessation group, although effects on moderate depressive conditions were not statistically significant. 

Meleis describes indicators of healthy transition as adaptive capacity and reduction of severe complications, even amid fluctuations.^16^^,^^17^ The elimination of severe depression suggests that the group functioned as a transition facilitator despite expected ups and downs during change.

Despite the high prevalence of depressive symptoms among participants, no statistically significant association was found between nicotine dependence levels and depression scores in this study. This finding contrasts with results from another Brazilian study that reported a higher frequency of depressive symptoms among smokers with high dependence levels according to the Fagerström Test.^18^ This discrepancy may be related to methodological differences, sample characteristics, or contextual factors influencing the relationship between smoking and mental health. Recent research reinforces the association between smoking and depressive disorders, showing that smoking tends to be more prevalent among individuals with depressive symptoms, highlighting the existence of complex mechanisms linking these conditions.^18^

The presence of this association underscores the importance of incorporating mental health care into smoking control and cessation strategies.^19^^,^^20^ An integrated approach that includes psychological support may increase program effectiveness and reduce relapse rates. Although there is a clear association between smoking and depressive symptoms, this study did not identify a significant correlation, possibly due to the small sample size, which limits statistical power. Moreover, the relationship between tobacco use and depression is complex and bidirectional: nicotine may temporarily relieve depressive symptoms, reinforcing use, while depression may precede or emerge during the cessation process.^22^^,^^25^

Although the small sample size, absence of a control group, and potential self-selection bias require caution in generalizing findings, this study contributes to the international literature by providing contemporary evidence on collective interventions at the interface between smoking and mental health. These results offer support for strengthening public policies and developing more precise care guidelines aligned with current demands for addressing smoking across populations.

Conclusion. This study demonstrated the positive impact of a group intervention guided by Afaf Meleis’ framework on reducing anxiety and nicotine dependence during treatment among smokers wishing to quit. It also contributes to advancing nursing scientific knowledge by validating the applicability of a theoretical framework in the field of substance dependence, consolidating smoking cessation as a complex situational transition requiring interventions beyond the biomedical model. In professional practice, the findings provide direct support for implementing systematized and personalized care protocols. The nurse’s role as a facilitator of support groups emerges as an autonomous and effective intervention strategy capable of strengthening self-efficacy and managing emotional symptoms predictive of relapse. Adherence to the group, combined with individuals’ intrinsic motivation and qualified professional support, demonstrated the effectiveness of the group approach as an integrative and personalized strategy for cessation support.

This research may contribute to strengthening public smoking control policies based on evidence that values articulated psychosocial and pharmacological interventions. This recommendation aligns with Meleis’ systemic perspective, which emphasizes that the sustainability of health transitions depends on recognizing and integrating multiple dimensions, promoting continuity of institutional and social support. Further investigations on the effectiveness of group therapies in treating nicotine dependence are essential, considering changes in consumption patterns and care systems, in order to strengthen cessation strategies and support evidence-based policies aligned with current international public health realities.
